# Phase I/II study on kilovoltage surface brachytherapy in conjunctival cancer: preliminary results

**DOI:** 10.3332/ecancer.2018.835

**Published:** 2018-05-15

**Authors:** Gustavo R Sarria, Gustavo J Sarria, Paola Fuentes Rivera, Mayer Zaharia, Solón Serpa, Mario Buitrago

**Affiliations:** 1 Radiotherapy Department, National Institute of Neoplastic Diseases (INEN), Lima 15038, Peru; 2 Ophthalmic Oncology Department, National Institute of Neoplastic Diseases (INEN), Lima 15038, Peru

**Keywords:** surface brachytherapy, kilovoltage, conjunctival carcinoma

## Abstract

**Introduction:**

In ocular conjunctival carcinoma after surgery, adjuvant treatment has a role and kilovoltage surface brachytherapy opens a new door for the range of therapeutic options.

**Materials and methods:**

Between October 2014 and June 2017, at the National Institute of Neoplastic Diseases (INEN) from Peru, 39 patients with squamous cell carcinoma of ocular conjunctiva, T1–T3, resected, were selected to receive adjuvant treatment. The portable accelerator of 50-kV INTRABEAM (Carl Zeiss Meditec) was used, after local anaesthesia and blocking of ocular muscles movement. The doses used were 18 Gy for patients with free margins and 22 Gy for positive edges, according to calculation of equivalent dose of 2Gy per fraction of 46 and 66 Gy, respectively, assuming a tumoural *α/β* ratio of 8 Gy. The prescription was done to 2 mm depth.

**Results:**

The median age was 69 years, distributed evenly between both genders, with a median follow-up of 12 months. The surgical margins were 59% free and 41% committed, with no difference between the institutions where the surgery was performed (*P* = 0.069). The median tumour size was 7 mm with 2 mm of invasion, 61.5% was T2 and 35.9% T1. The mean time between surgery and irradiation was 1.5 months, 23.1% of patients developed grade I toxicity of spontaneous resolution, without evidence of greater degree in any case. The dose had no statistical relationship with toxicity (*P* = 0.533). One-year disease-free survival was 96.7%.

**Conclusions:**

Kilovoltage surface brachytherapy is an applicable and reproducible tool in the treatment of squamous cell carcinoma of ocular conjunctiva. The administered doses are well tolerated by patients with low levels of acute toxicity. Longer follow-up is needed to establish disease control rates and late toxicities.

## Introduction

Conjunctival squamous cell carcinoma is the second most frequent malignant ocular tumour [1, 2, 3]. The bulbar conjunctiva is where this disease most frequently presents itself, and it may have a poor prognosis if it is not diagnosed and treated early. Newton *et al* [4] found that the rate of conjunctival squamous cell carcinoma decreases by approximately 49% for every 10% increase in latitude. For example, Uganda exhibits 12 new cases per million inhabitants per year, whereas England has a rate of 0.2 new cases per million inhabitants per year [4, 5]. In studies conducted in the United States, the rate was 0.3 patients per million inhabitants per year [4].

Amongst the Peruvian population, according to the available data from the Lima Metropolitan Cancer Registry 2010–2012, there were 157 new cases in this selected period, 83 in males and 74 in females, with the population over 70 years of age being the most affected. There are currently no national statistics [6].

Currently, the primary operation is surgical resection, which identifies various adjuvant tools such as cryotherapy, topical chemotherapy and radiotherapy in its various forms (external radiotherapy and/or brachytherapy), to improve disease-free survival according to various reports [1, 7]. Originally, brachytherapy was administered with plates of radioactive isotopes, which remained in contact with the surface that was to be treated for different times, depending on the rate of the emitted dose and required depth [8].

This study seeks to determine the usefulness of single-dose surface kilovoltage brachytherapy, analysing the applicability, toxicity and disease control rates which it could provide.

## Objectives

### Main objectives

To establish the rate of acute complications in patients diagnosed with conjunctival squamous cell carcinoma, T1–T3, who are undergoing kilovoltage surface brachytherapy.

To establish the optimum tolerable dose of treatment in patients diagnosed with conjunctival squamous cell carcinoma, T1–T3, undergoing kilovoltage surface brachytherapy.

### Secondary objectives

To discover the 5-year disease-free survival rate in patients diagnosed with ocular conjunctival squamous cell carcinoma, T1–T3, undergoing kilovoltage surface brachytherapy.

## Hypothesis

The rate of acute complications in patients with a diagnosis of conjunctival squamous cell carcinoma, who were operated on and who underwent kilovoltage surface brachytherapy, is acceptable.

The optimal tolerated single-fraction dose of treatment is equivalent to 46 Gy and 66 Gy, according to the status of surgical margins, at fractions equivalent to 2 Gy, by applying the linear-quadratic model.

The 5-year disease-free survival rate is comparable with the historical reports of treatment with external radiotherapy.

## Materials and methods

Between October 2014 and June 2017, 39 patients diagnosed with conjunctival squamous cell carcinoma T1–T3, who underwent surgical resection, but not topical chemotherapy nor any other nonradiotherapy treatment, received adjuvant therapy with surface brachytherapy on the operating table in one single application.

The patients’ position was set by placing thermoplastic masks, fenestrated on the patient’s ocular region. Eye movements were limited by blocking the extrinsic musculature of the eyeball, and the tissues were exposed with a blepharostat, with prior local surface anaesthesia with drops. The portable 50-kV INTRABEAM accelerator (Carl Zeiss Meditec) was used, with Flat Applicators with a variable diameter of between 1 and 2cm, depending on the characteristics of the injury treated.

The doses prescribed were 18 and 22 Gy, according to the status of surgical margins; free or compromised, respectively. A radiobiological equivalent of 2 Gy/fraction was established, assuming a tumoural *α*/*β* ratio of 8 Gy. This is generally the value related to head and necksquamous cell carcinoma. The dose was calculated using the formula:

$$\mathrm{EQD}2=D\;\cdot \;\frac{d+(\alpha /\beta )}{2\;\mathrm{Gy}+(\alpha /\beta )},$$

based on the linear-quadratic model, and equivalent values of 46.8 and 66 Gy at 2 Gy/fraction were obtained. The prescribed dose was administered at a depth of 2 mm, in relation to the thickness of the ocular structures.

The toxicity was evaluated according to the Common Terminology Criteria for Adverse Events (CTCAE) v4.03.

## Ethical considerations

The application of treatment was under the express authorisation of the patient by signed informed consent, with full knowledge of potential adverse effects from the use of radiation, which is recorded in the clinical history.

## Statistical analysis

A descriptive analysis of the data was carried out through frequencies, percentages and summary measures (average, media and range). Using the Chi-square test, the association amongst qualitative characteristics was evaluated, using Yates’ correction where necessary.

Differences between groups with a qualitative characteristic with reference to quantitative characteristics were evaluated with the *t*-test for independent samples (by a normality test of these characteristics), or its corresponding nonparametric test. A *P*-value <0.05 was considered for an association or significant difference. The statistical analysis used R software [R Core Team (2017). R: a language and environment for statistical computing. R Foundation for Statistical Computing, Vienna, Austria. URL: https://www.R-project.org/].

## Results

**Figure 1.**
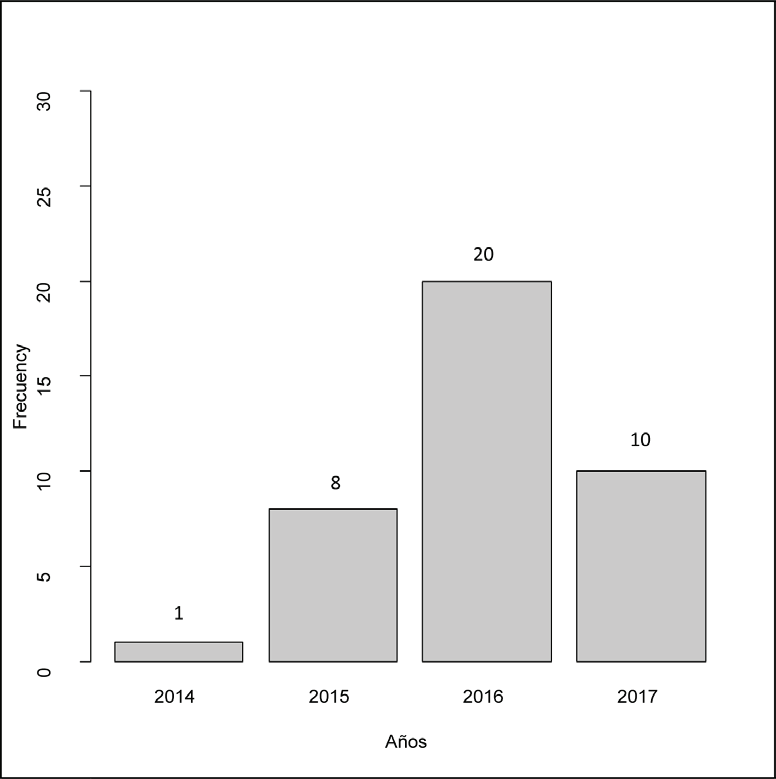
Frecquency per years of IB applications.

**Figure 2.**
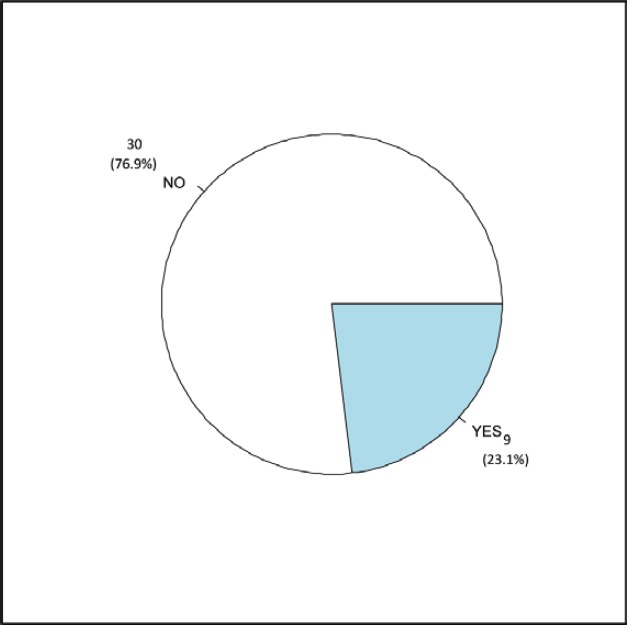
Complications rate.

**Figure 3.**
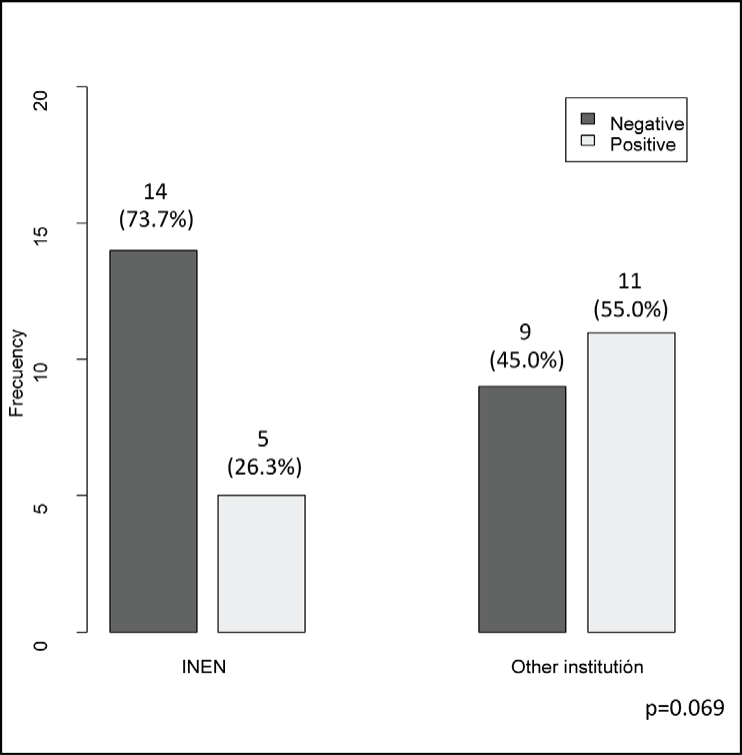
Borders status (negative/positive) according to surgery place.

**Figure 4.**
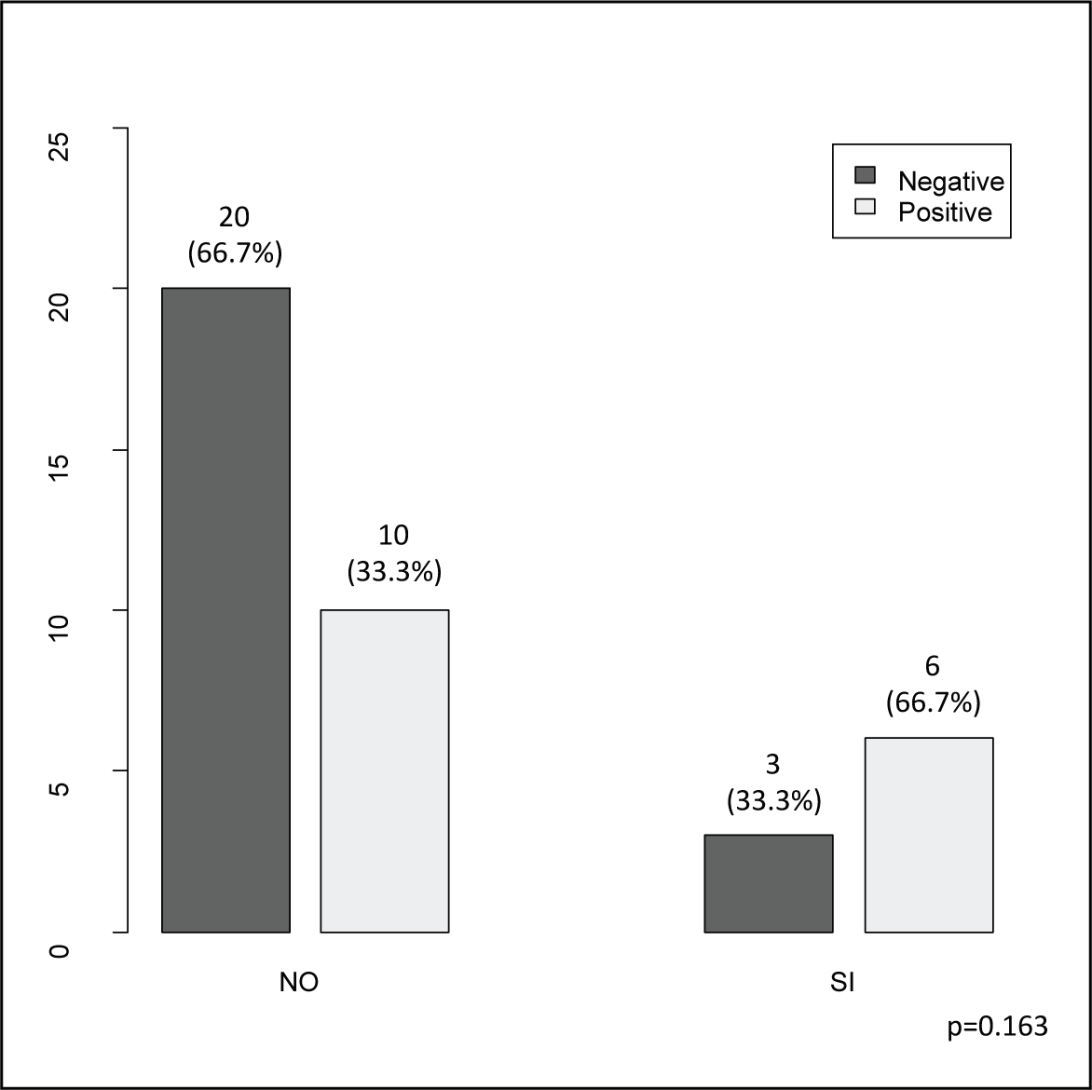
Borders status (negative/positive) according to the presence of complications (no/yes).

**Figure 5.**
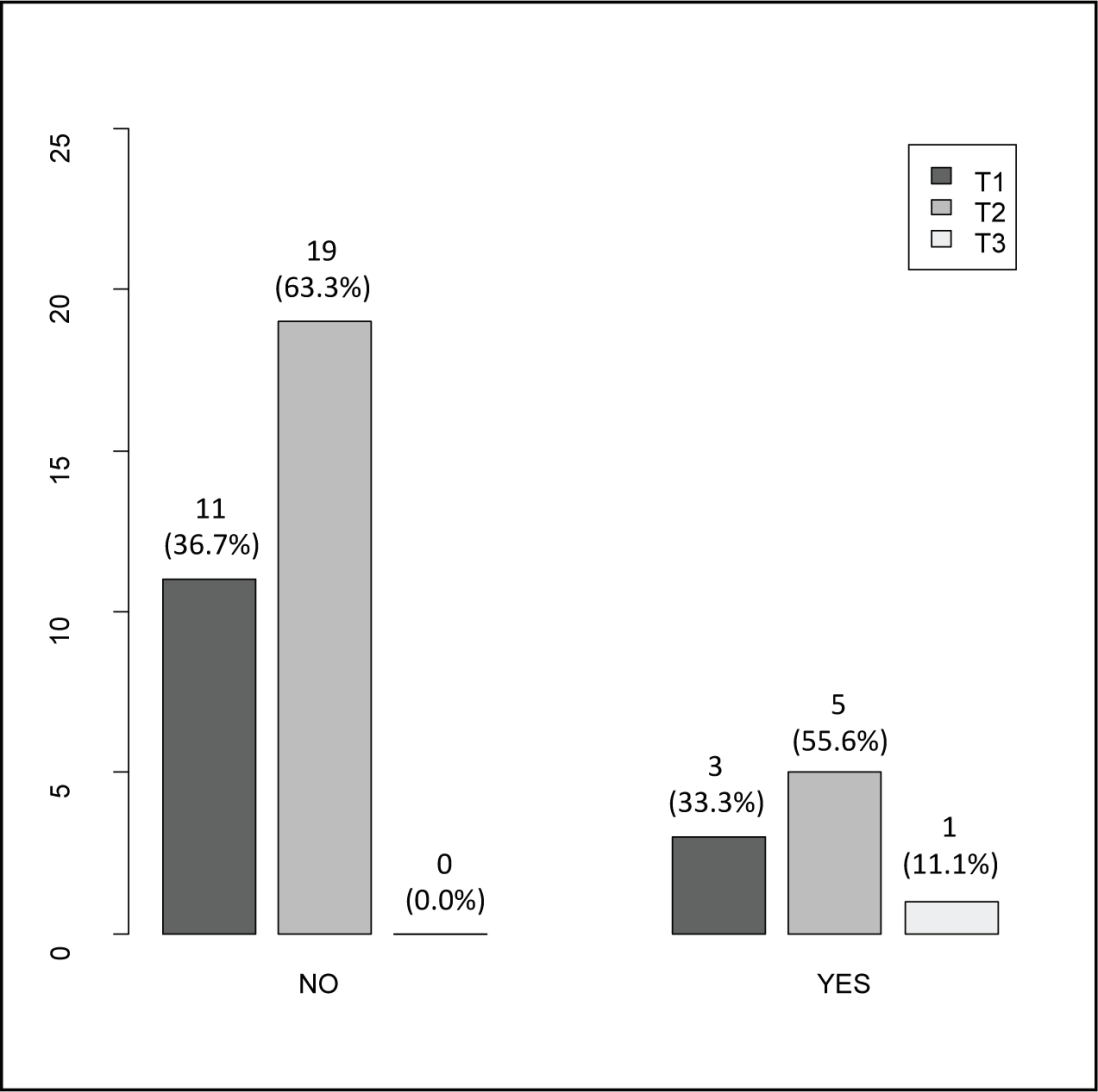
T stage (T1/T2/T3) according to the presence of complications (no/yes).

**Figure 6.**
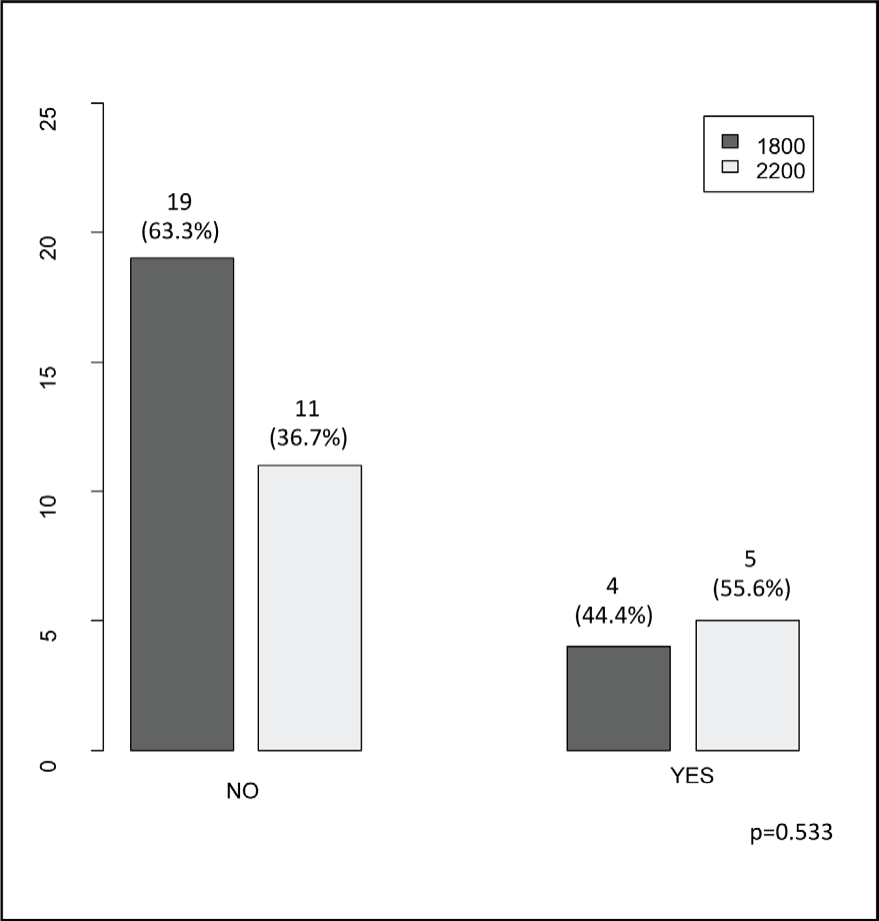
Dosis (1800 cGy/2200 cGy) according to the presence of complications (no/yes).

The clinical (Table 1), treatment (Table 2) and pathological characteristics (Table 3) of 39 patients with conjunctival squamous cell carcinoma are described. All patients were alive at the time of evaluation. The distribution by *T* classification was 35.9% for T1, 61.5% for T2 and 2.6% for T3. Frequency by years of treatment with INTRABEAM is shown in Figure 1 (recorded until June 2017). Nine patients (23.1%) with acute complications were documented (Figure 2). All patients were grade G1 and spontaneous resolution within the first-month post-treatment. In all cases, there was erythema or mild itching. No late toxicity has been found so far. It was found that the status of the margins and the place of surgery are not associated (Figure 3). Figure 4 shows that the status of margins and complications are independent; their association with the T stage could not be evaluated (Figure 5), nor was an association between the dose and the complications observed (Figure 6). In the group of patients with no complications, the average tumour size was 7.62 mm (range 2–20 mm) and in the group with complications, the size was 8.39 mm (range 1.5–20 mm). A significant difference was not found between the two groups in terms of size (*P* = 0.701).

**Figure 7.**
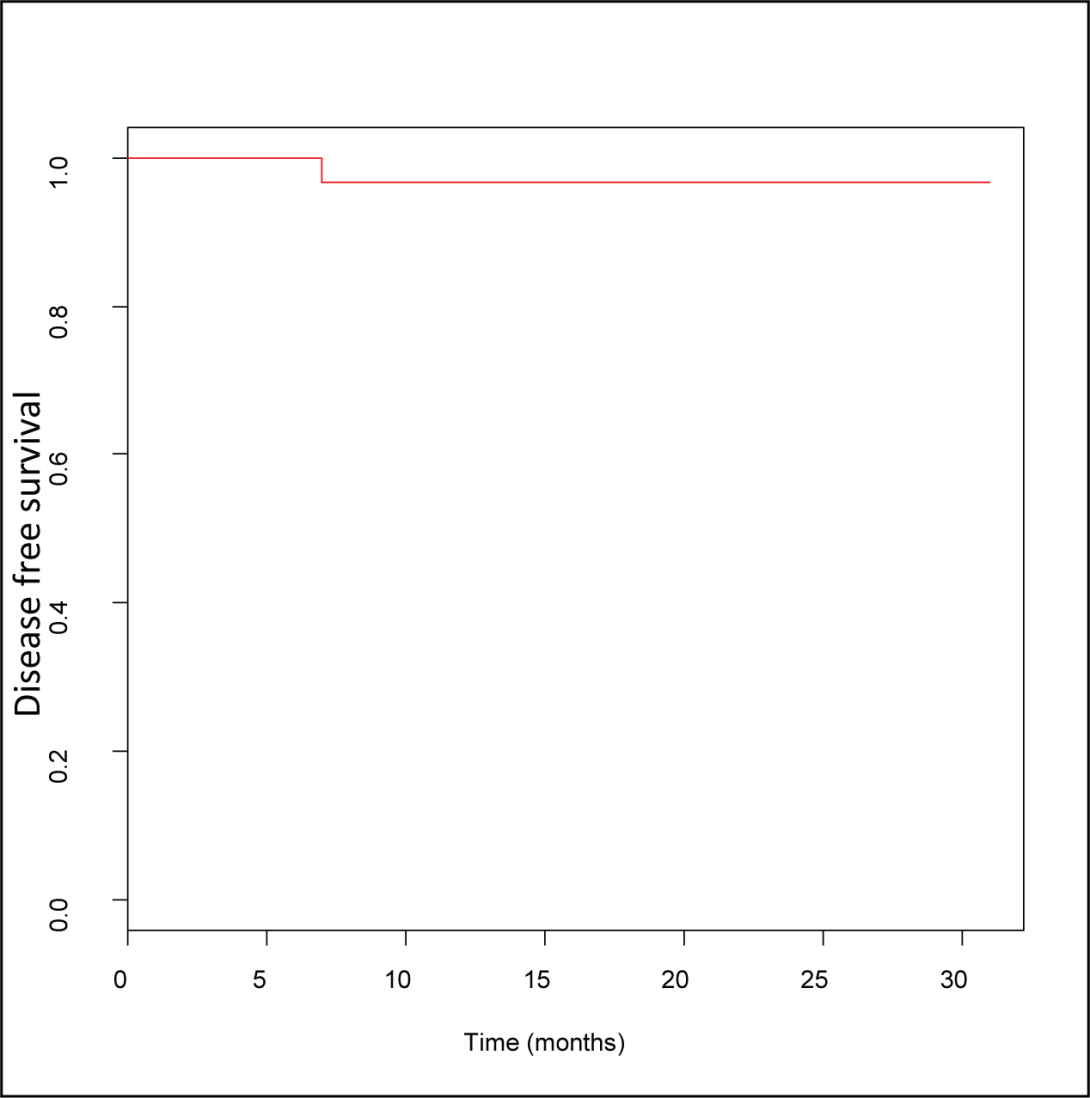
Estimated disease free survival curve.

The median follow-up time was 12 months, with a minimum of 3 months and a maximum of 31 months. Only one patient, with compromised surgical margins, presented a recurrence of disease during follow-up 7 months after having undergone treatment. One-year disease-free survival was 96.7%, at the time there being no recurrence of disease in the patients with a longer follow-up time (Figure 7).

## Discussion

In health systems such as that in Peru, the administration of shortened treatment is introduced as an attractive alternative to reduce costs, without affecting the patients’ quality of care. In addition, the limited accessibility to radiotherapy services, both in Peru as well as in the rest of the region, and the prolonged residence of patients far from their home, represent an additional cost that is not financed by the health system, but rather by the patient or family themselves [9].

The need for adjuvant therapy in conjunctival squamous cell carcinoma is imperative, given the results of disease control at the international level, compared to surgery alone. Galor *et al* [7] published a retrospective series of 389 patients who underwent local resection of ocular surface carcinomas in 2012, establishing risk factors for local recurrence. It presents figures of 10% recurrence in the first year follow-up and 21% at 5 years. It Identifies that T2 and T3 have a greater probability of recurrence than T1 using (T2/T1 hazard ratio [HR], 2.05 [*P* = 0.04]) T3/T1 HR, 2.31 (*P* = 0.07), the Tarsus HR compromise, 4.12; *P* = 0.007. Treatment with adjuvant cryotherapy decreases the rate of recurrence significantly in patients with compromised margins, HR, 0.51; *P* = 0.03, bringing the value of resected neoplasms with free margins to around 10% control in 5 years [7]. In successive series such as that of Laskar *et al* [10], it is demonstrated that patients subject to resection with free margins can reach recurrence rates up to 33%, whilst patients with compromised margins reach up to 56%. In their work, they reported treatment with strontium plates at variable dosage, taking averages of 44 Gy, in daily doses of 4 Gy, and disease-free survival rates of 90.9% at 5 years. No patient in their publication had a toxicity greater than or equal to grade II [10]. Kearsley *et al* [11] in 1988 reported interesting results for the use of kilovoltage at variable dosage between 45 to 50 Gy in 8 to 15 fractions and a single fraction of 10 Gy, without recurrences in the treated patients, with follow-up periods exceeding 10 years and grade I early and late toxicities [11]. In our institute, retrospective figures of 5-year disease-free survival occurred around 78.8 to 82.3%, at the stage of post-surgery adjuvant external radiation, with daily doses of 2 Gy/fraction and variable totals between 45 and 66 Gy, according to the status of surgical margins.

The application of one single session of surface brachytherapy greatly decreases the potential errors of configuration and positioning, amongst others, which are subject to variation by daily treatments, achieving equivalent doses for disease control that have even greater biological value, given its nature [12].

The procedure performed proved to be highly reproducible for the treated patients. The minimal invasiveness to achieve a stable position for the eyeball is remarkable, in a similar fashion to that reported by Semenova and Finger [13]*.*

Acute toxicity has been acceptable, within grade I, according to the criteria of the CTCAE v4.03 [14]. Every case had a spontaneous resolution within the first-month post-treatment, without the need for medical intervention. Late toxicity is not evident at this time. These findings correlate with the descriptions in the literature [10, 11], achieving dose escalation suitable for disease control. The use of kilovoltage allows for a more predictable dosimetry compared to radioactive isotopes and eliminates the unnecessary exposure of patients and health personnel to ionising radiation. To date, our Institution has replaced the use of isotopes for surface treatment, in line with the international trend towards migration to more secure systems of treatment, which allow for versatility in pathology management of other anatomical regions [15].

One constraint is that the study is being carried out in only one institution. The support of other centres on a global level would deliver more legitimacy and statistical validity to the obtained results.

The preliminary results of disease-free survival are encouraging, taking into account the recurrent nature of this entity. However, it is premature to draw conclusions regarding the rates of control, given the short follow-up time to date. A long-term follow-up is necessary in order to determine the real impact value of this form of treatment, such as the toxicity that could present itself over time.

## Conclusions

The use of kilovoltage in the management of conjunctival squamous cell carcinoma is reproducible, viable and safe, according to the experience of our Institution. Acute toxicity, as a consequence of the treatment, is acceptable when compared to the potential benefits offered by this method. The support of other international centres in this investigation is important to reach a greater case volume and improve the strength of this study. A greater follow-up period is needed to determine long-term late toxicities and disease-free survival rates in the group of patients who were analysed.

## Conflicts of interest

The participants stated that they had no conflicts of interest.

## Figures and Tables

**Table 1.** Clinical features.

	*N*	%
Age, years		
Average/median/range	63.64/69/[29–87]	
Gender		
Female	19	48.7
Male	20	51.3
Side		
Right	18	46.2
Left	21	53.8

**Table 2.** Treatment features.

	*N*	%
Surgery type		
Biopsy	4	10.3
Local wide excision	35	89.7
Place of surgery		
INEN	19	48.7
Other institution	20	51.3
Time between surgery and IB, months		
Average/median/range	1.821/1.5/[0–5]	
Dosis cGy		
1800	23	59.0
2200	16	41.0

**Table 3.** Pathological features.

	*N*	%
Borders		
Negative	23	59.0
Positive	16	41.0
Size, mm		
Average/median/range	7.795/7/[1.5–20]	
T		
T1	14	35.9
T2	24	61.5
T3	1	2.6
Depth, mm (*N* = 19)		
Average/median/range		2.2/2/[0.1–6]
